# Fabrication of three-dimensional suspended, interlayered and hierarchical nanostructures by accuracy-improved electron beam lithography overlay

**DOI:** 10.1038/s41598-017-06833-5

**Published:** 2017-07-27

**Authors:** Gwanho Yoon, Inki Kim, Sunae So, Jungho Mun, Minkyung Kim, Junsuk Rho

**Affiliations:** 1 Department of Mechanical Engineering, Pohang University of Science and Technology (POSTECH), Pohang, 37673 Republic of Korea; 2 Department of Chemical Engineering, Pohang University of Science and Technology (POSTECH), Pohang, 37673 Republic of Korea; 3 National Institute of Nanomaterials Technology (NINT), Pohang, 37673 Republic of Korea

## Abstract

Nanofabrication techniques are essential for exploring nanoscience and many closely related research fields such as materials, electronics, optics and photonics. Recently, three-dimensional (3D) nanofabrication techniques have been actively investigated through many different ways, however, it is still challenging to make elaborate and complex 3D nanostructures that many researchers want to realize for further interesting physics studies and device applications. Electron beam lithography, one of the two-dimensional (2D) nanofabrication techniques, is also feasible to realize elaborate 3D nanostructures by stacking each 2D nanostructures. However, alignment errors among the individual 2D nanostructures have been difficult to control due to some practical issues. In this work, we introduce a straightforward approach to drastically increase the overlay accuracy of sub-20 nm based on carefully designed alignmarks and calibrators. Three different types of 3D nanostructures whose designs are motivated from metamaterials and plasmonic structures have been demonstrated to verify the feasibility of the method, and the desired result has been achieved. We believe our work can provide a useful approach for building more advanced and complex 3D nanostructures.

## Introduction

Nanoscale fabrication techniques play an important role not only in current semiconductor industries, but also in state-of-the-art academic researches such as nanomaterials, nanoelectronics and nanophotonics^1–4^. The methods are typically divided into two categories, i.e. top-down and bottom-up methods^5^. Top-down methods, including different kinds of lithographic techniques based on high energy photons, electrons and nanoscale molds, are useful to define rigorously designed structures^6^. On the other hand, bottom-up methods, which are usually based on chemical reactions in the form of solutions, contain more randomness than top-down techniques^7–14^.

Electron beam lithography (EBL), as one of the lithographic techniques, is widely used for making nanostructures owing to its high-resolution patterning capability without any masks^15–19^. Basically, it gives one layer of nanostructures from one unit process including exposure, development, deposition and lift-off steps, but we can also fabricate three-dimensional (3D) nanostructures by repeating this unit process on the same position of the substrate, i.e. stacking the individual two-dimensional (2D) layers^20^. At this moment, alignment accuracy among the stacked layers determines the overall 3D structuring quality because the EBL overlay process usually contains quite large errors (around micrometer scale). To build elaborate 3D nanostructures using EBL overlays, reducing the alignment errors is certainly the most critical factor.

Here, we suggest a simple approach to minimize alignment errors in EBL overlay processes. Newly designed alignment marks and calibrators are our strategies. Alignment marks should preserve their alignment spot consistently during the process, but there is no standard configuration of EBL alignment marks. Widely used cross-type and circle-type alignment marks can easily lose their alignment spot due to other fabrication steps such as photoresist development and pattern transfer (e.g. lift-off). We have designed new alignment marks, which can improve alignment errors by reducing proximity effects near the center of the marks. Unique calibrators, (e.g. nano-ruler) have also been applied to compensate for the mechanical errors of the stage movement. As a result, we could achieve sub-20 nm EBL overlay accuracy and realize complex 3D nanostructures motivated by metamaterials as instances. We also believe that the proposed alignment mark configurations and calibrators can be a useful guideline to fabricate a variety of arbitrary and complex 3D nanostructures based on EBL overlay processes.

## Results

### Misalignment in conventional EBL overlay process

**Figure 1:**
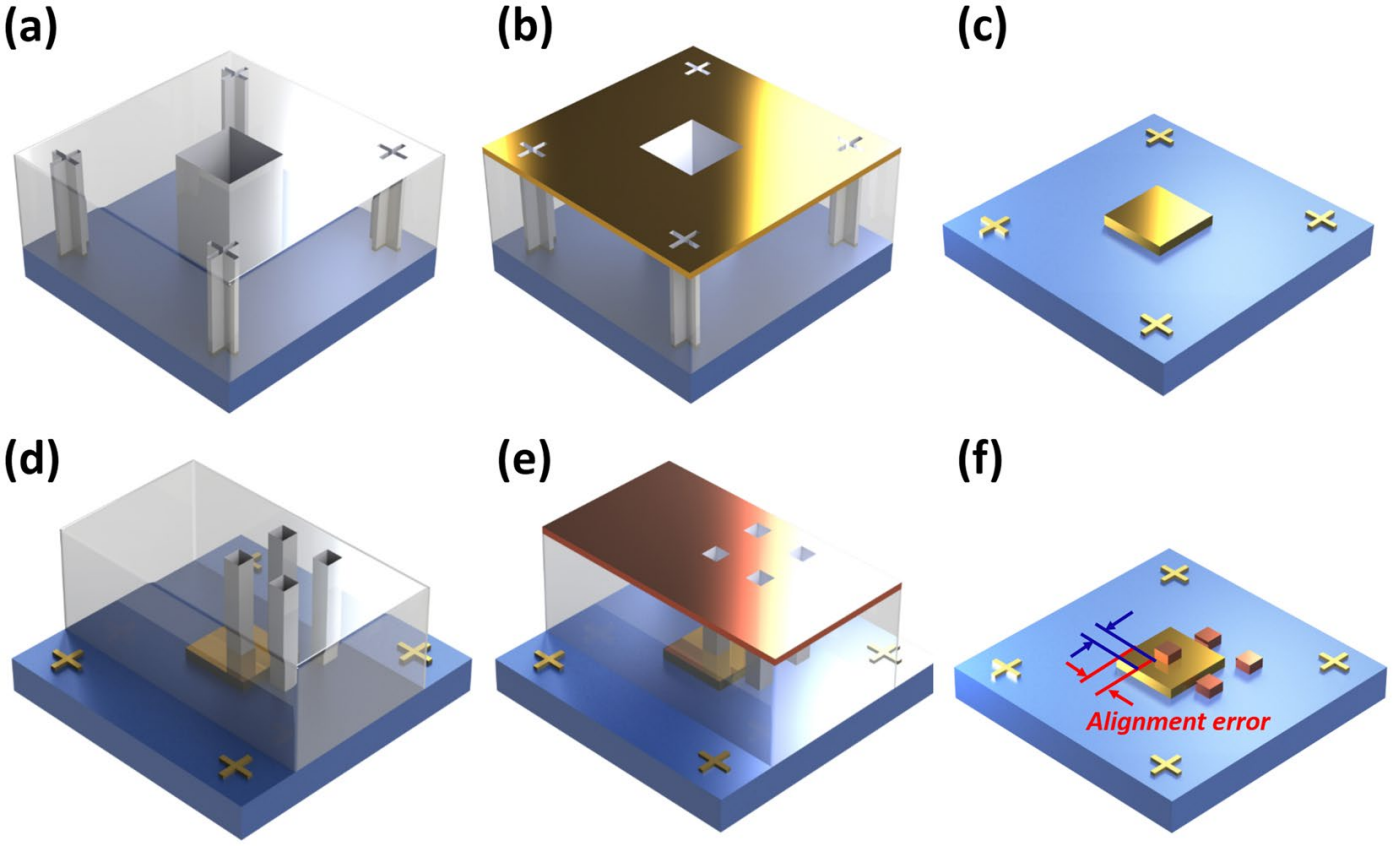
Schematic of conventional EBL overlay process. (**a**) Alignment marks and first layer patterning process on the copolymer (MMA) and polymethyl methacrylate (PMMA) bilayer resists. (**b**) Material deposition and lift-off process. If one wants to use different materials in patterned area and alignment marks, or to execute multiple EBL overlay processes, the alignment marks should be blocked to prevent the material deposition during the first layer patterning. (**c**) Fabricated alignment marks and first layer patterns. (**d**) Subsequent EBL overlay process (the second layer patterning) with the pre-defined alignment marks. Since we have to find and use alignment marks using electron beam scanning, the alignment marks area uncovered by resists are favorable to give clearer observation in scanning electron microscope (SEM) mode. For this purpose, we removed the PMMA covering alignment marks before the second EBL process. Particularly, this conventional overlay process contains alignment errors caused by mechanical errors (e.g. stage movement). (**e**) Material deposition for the second layer. (**f**) Final result with a considerable misalignment.

The basic idea of EBL overlay process is stacking individual 2D layers with multiple exposures. (Fig. 1) In order to understand the overlay process, it is necessary to be aware of the details of the electron beam exposing steps. The first step is inserting the pattern information into the EBL system. Then, we have to divide the pattern into unit cells and set the dose we will expose on each cell. The optimal dose value varies according to the shape and density of the patterns. It means that some control experiments are inevitable to find out the optimal dose value to make appropriate structures. One of the important concepts of the EBL process is a “chip” (or “write window”) meaning an area where an electron beam can scan without any stage movement. Only the current in the electromagnetic lens changes for scanning the area in a single chip. In the case of several chips, the stage should move between each chip. This process is called stitching, which means alignment among chips on the same layer. If we use the chip array to cover the whole pattern, stitching errors due to stage movement must be compensated for as they always introduce some errors. General EBL systems provide several chip sizes, e.g. 150 × 150 μm^2^, 300 × 300 μm^2^ and 600 × 600 μm^2^. Depending on the chip size, resolution and alignment accuracy also change. When we use 600 × 600 μm^2^ chips, error in the chip itself such as distortion should be corrected before the exposing step. Usually, these compensation functions have to be calibrated by finding the offset values of the x-direction, y-direction and rotation before the overlay process. After doing a number of repeated calibrations, the stitching error, which is different to the overlay error, can be reduced to less than 20 nm.

Alignment marks are necessary for the EBL alignment process. It is similar to that of photolithography. When we define a certain pattern on the bare substrate, alignment marks are not necessary. However, in the case of a repeating patterning on the previously fabricated layers, a relative position between each pattern has to be seriously considered. We can imagine two 2D coordinates, i.e. the substrate and EBL system. Although most systems provide a relative coordinate of the substrate based on the substrate holder, usually these two coordinates are mismatched without additional alignment processes even if we attach our substrate perfectly on the holder. It is even more obvious when it comes to nanoscale. This is the reason why the alignment step is necessary in EBL overlay processes. To match the coordinates, we need to accurately overlap at least two points of each coordinate. If we use only one point for each coordinate, rotation of each coordinate cannot be fixed. At this time, alignment marks are used to indicate the aforementioned points on the substrate. For example, if we locate a certain corner of the alignment marks at (0 μm, 0 μm) and (100 μm, 100 μm) based on the substrates coordinates, we need to find the two points using the SEM function of the EBL system. This information is used to set the coordinates of the EBL system which is actually used for electron beam exposing. Without alignment marks, there is no way to know where (0 μm, 0 μm) and (100 μm, 100 μm) are located through SEM.

The second EBL process is identical to the first EBL process except that it needs an additional alignment step. We should match a coordinate of the stage saved in the system to a coordinate of the real stage. Usually, two cross-type alignment marks located at a certain distance apart are used to compensate the alignment errors. Using the SEM function, we have to find alignment marks manually and match the reference points of the marks to the virtual alignment marks shown on the display that represent a relative position between the electron beam and the stage. After designation of the reference points, if we use an automatic alignment function the stage will move between two alignment marks repeatedly to correct the origin position and the rotational errors of its coordinate. So far, the general EBL overlay process has been introduced.

### Approaches for improving EBL overlay accuracy

Unfortunately, after the standard aforementioned process, most EBL systems show imperfect alignment between the layers. The errors are results from two major defects, i.e. alignment marks and the sample stage. The error of alignment marks originates in the difference between the designed alignment marks and the fabricated alignment marks. Due to the proximity effect, the actual alignment marks slightly expand, and their edges become blunt^21, 22^. This expansion affects the position of the alignment spot of the mark, and the blunt edges increase alignment uncertainty at high magnification. The other defect of the stage is related to mechanically induced bias errors, hence, the bias errors vary depending on the EBL systems. However, in our experience, the errors occuring from stage movement seem to be constant and larger than the errors from alignment marks. The following explains how we can control the two errors above.

**Figure 2:**
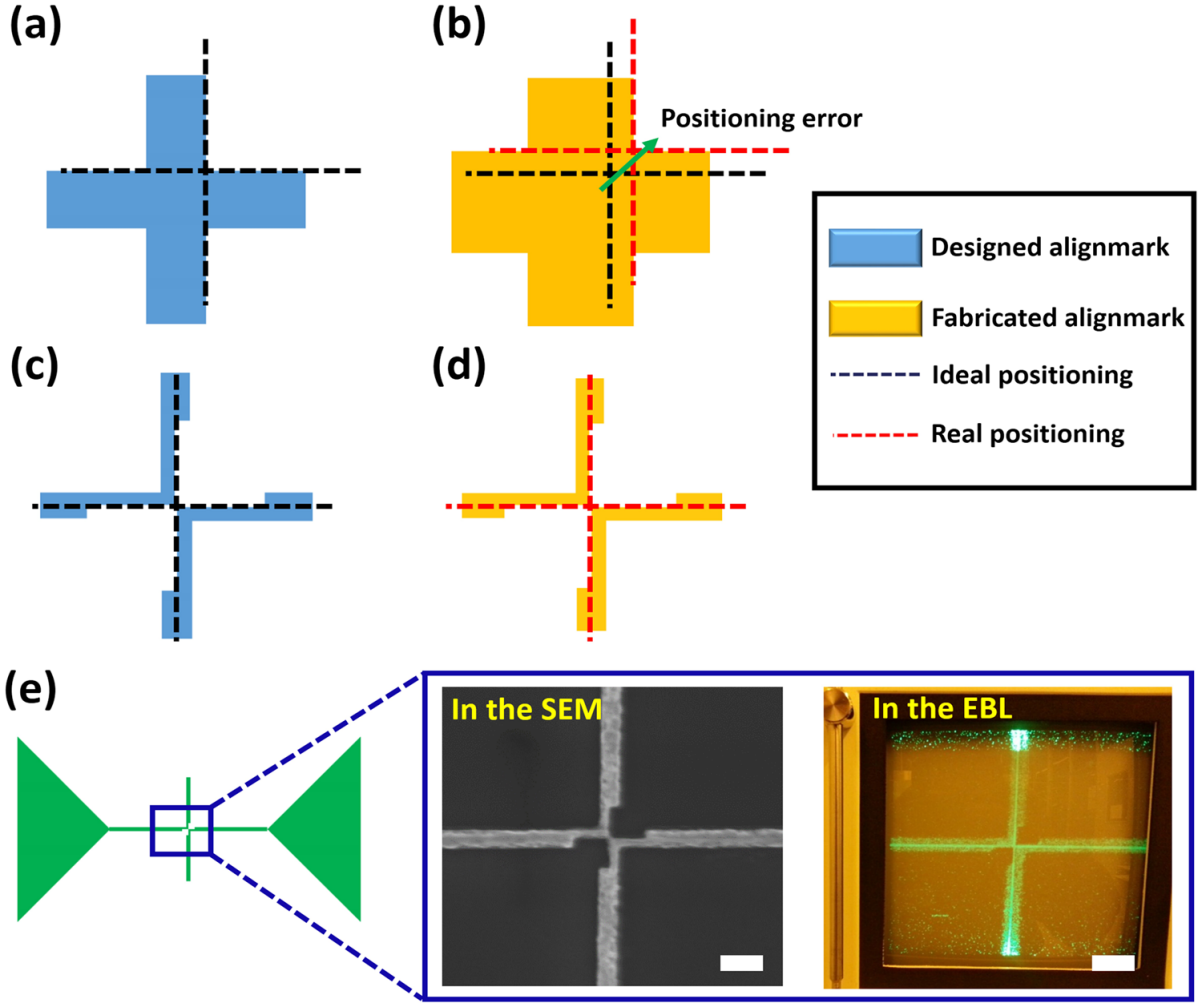
Illustration of the idea on improving alignment and corresponding alignment marks. Since conventional alignment marks can be easily deformed by the proximity effect due to their geometry, complex 3D nanostructures could not be obtained easily. Therefore, new alignment marks are designed to achieve improved overlay accuracy. (**a**) Blue mark indicates designed marks and the black dashed line shows the ideal alignment position. (**b**) Yellow mark indicates fabricated marks and the red dashed line shows the actual alignment position. The errors come from the discrepancy between the designed mark and fabricated mark. The discrepancy results from a proximity effect of the electron beam. (**c**) Newly designed alignment mark. (**d**) Fabricated alignment mark. Because the alignment mark is composed of two bracket-shape structures and the structures meet at a point, the alignment mark is insensitive to the proximity effect. (**e**) Whole alignment mark design. Large triangle patterns are used to find the alignment mark easily and the center part is used for alignment. SEM image of the mark and a real picture of the screen during alignment steps in EBL mode. During scanning the alignment mark, we manually adjust the center point of the fabricated mark and virtual alignment mark shown as a solid green line. Scale bars represent 100 nm.

To maintain constant alignment spots against the proximity effect, we modify conventional alignment mark designs. (Fig. 2) Usually, the conventional design has a cross or circle shape which is impossible to achieve consistency against the proximity effect. (Fig. 2(a)) After a few attempts, we conclude that separate structures whose edges meet at a point can present acceptable results owing to their geometrical characteristics. (Fig. 2(b)) Although this newly designed alignment mark is affected by the proximity effect, we can easily find the original alignment spot since it lies on the center intersection of four nanobar structures. We also shrink the alignment mark to have 50 nm of the critical dimension to minimize the proximity effect. Since it has sufficiently sharp edges, we can conduct the alignment at the 90,000 magnification which is the maximum value of our EBL system. Surrounding thicker rod structures hold the center part, and microscale triangles are useful when we find the alignment mark using the SEM function.

**Figure 3:**
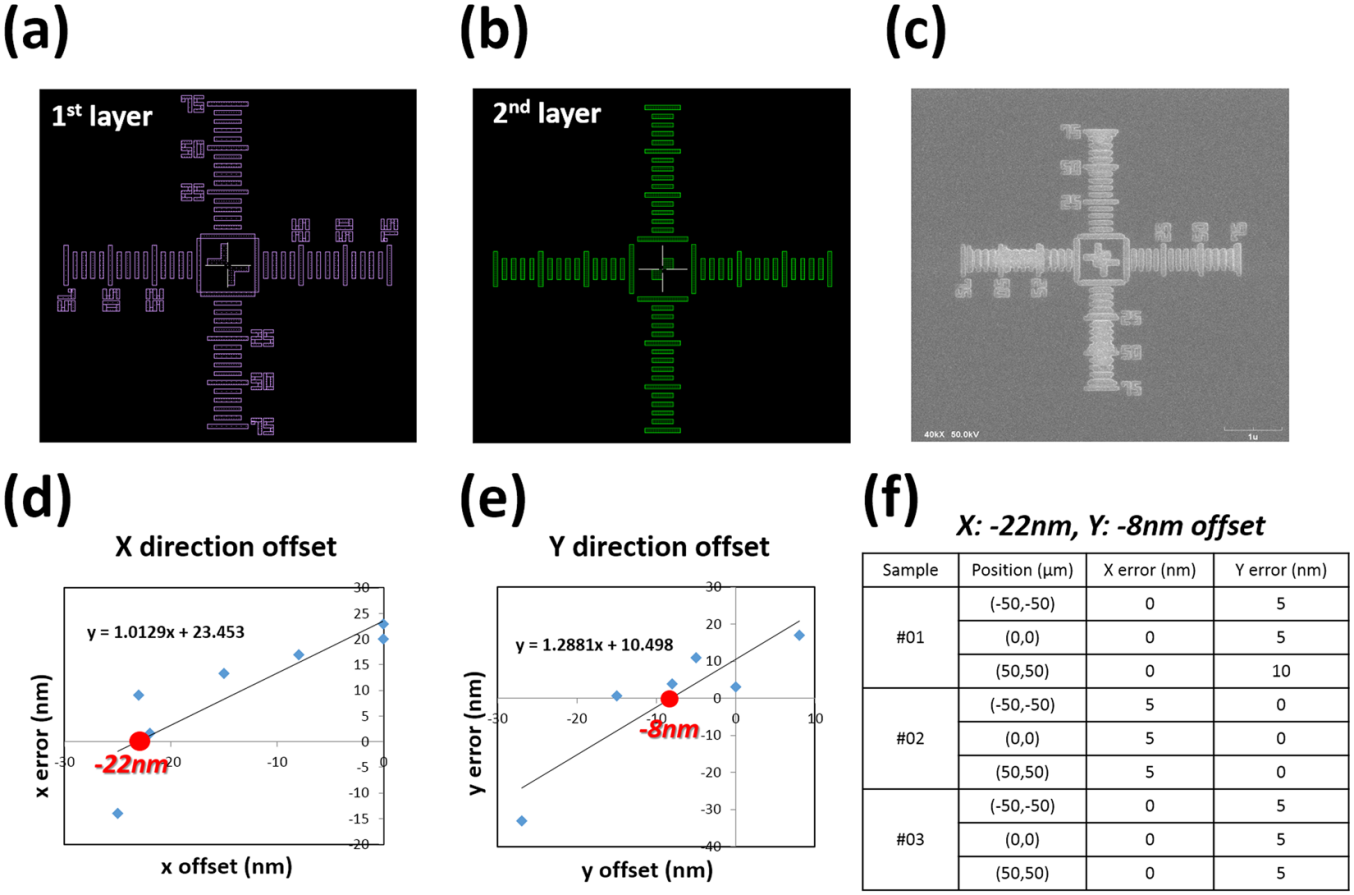
Alignment calibrator (nano-ruler). In order to compensate for alignment errors caused by mechanical errors (e.g. stage movement) we design a calibrator and measure the alignment errors. (**a**,**b**) The calibrator of the first and the second layer, respectively. (**c**) SEM image of a fabricated calibrator. The calibrator or ruler is used to measure alignment errors and each scale on the ruler represents 50 nm. After making the first layer calibrator structures, we can fabricate the second pattern on top of the first calibrator. If there are no alignment errors, then the second patterns should fit onto the first pattern exactly. Thus, by observing the fabricated patterns, we can find the proper offset to compensate for the alignment errors induced by mechanical errors. (**d**,**e**) Statistical results of the translational errors. By applying offset properly into the mask (CAD pattern) design, we can find the intrinsic alignment errors that occur from the EBL system. (**f**) Error data set after imposing the optimum offset. We test three samples and three alignment processes are done at different positions on each sample. Measured errors are sub-20 nm.

To eliminate the mechanical errors of the stage, an additional structure, called calibrator, is inserted to compensate for the unwanted misalignment factors. (Fig. 3) Although these obstructions can arise from any mechanical parts, most of them come from the backlash errors of the stage. Before starting a 3D structure fabrication, we can measure the mechanical errors using the calibrators. A ruler-shaped mark is used for the calibrator similar to the alignment marks defined in the first EBL step. The similar, but slightly smaller, square patterns will be drawn on the previous one through EBL overlay step. After fabrication of the second calibrator, any alignment errors will be detected through SEM. The errors can be adjusted by giving offset in the pattern design. Particularly, in this work, the calibrator is used to measure translational alignment errors.

**Figure 4:**
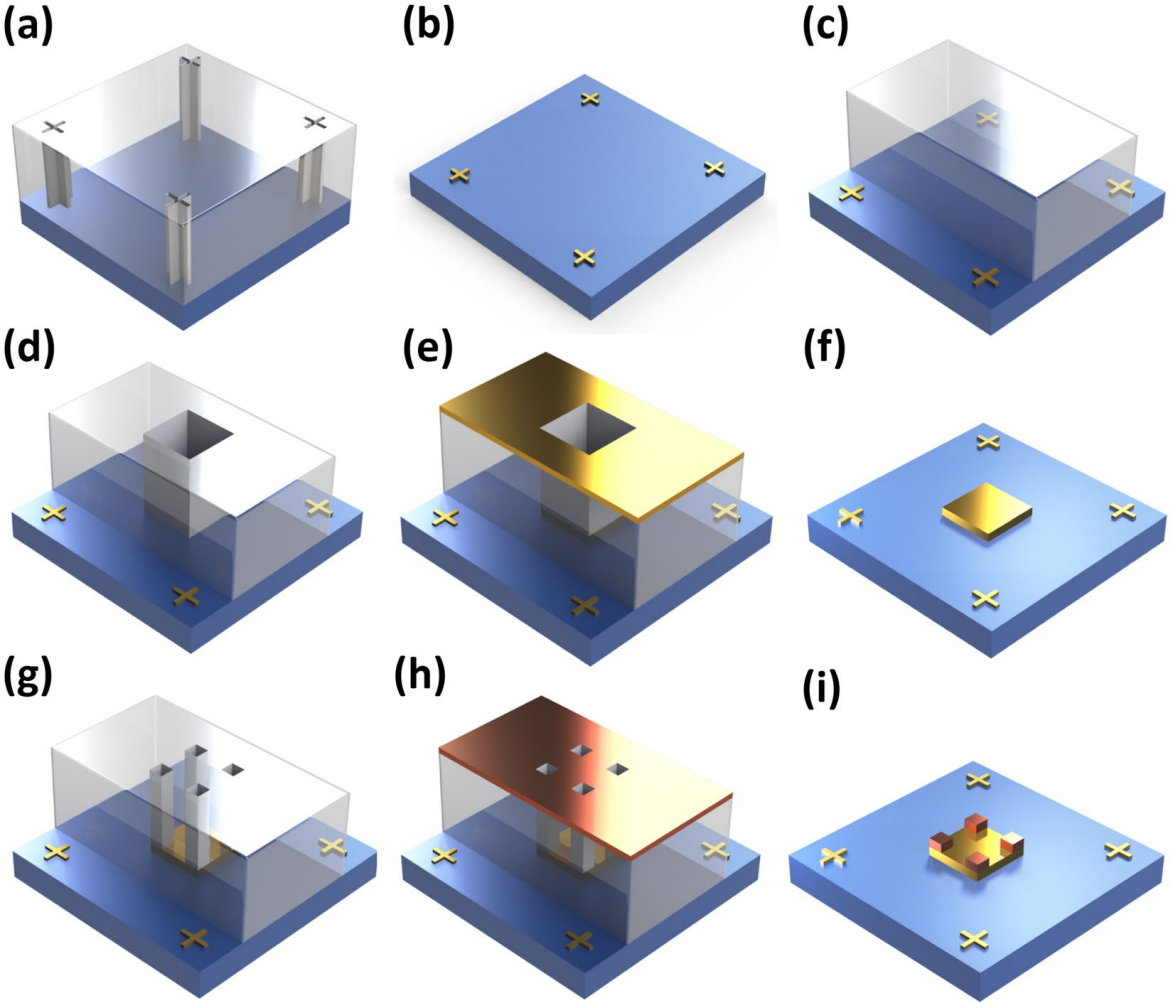
Flow chart of the modified EBL overlay process for improving accuracy. (**a**) Alignment marks patterning on MMA/PMMA bilayer resists. (**b**) Fabricated 50 nm thick gold alignment marks. (**c**) PMMA spin coating and removing resist on the alignment marks. (**d**) First layer patterning with the first alignment process. In the first layer patterning, alignment errors caused by mechanical errors (e.g. stage movement) are involved. (**e**) Material deposition for the first layer patterns. (**f**) Fabricated first layer pattern. (**g**) Second layer patterning with the second overlay process. As expected, in the second layer patterning, the same intrinsic alignment errors also occur. However, because the intrinsic errors are fixed in successive exposure steps, the alignment errors between the first and second layer are sub-20 nm. (**i**) Fabricated two-layered stacked 3D nanostructures. Through the continuous EBL overlay processes, we can build any arbitrary and complex 3D nanostructures within the overlay tolerance ranges.

Although the calibrator can detect the mechanical errors of the stage, we have invented a clever way to directly compensate for such mechanical errors. (Fig. 4) We found that the mechanical errors are uniform in a certain sample whenever we use same alignment marks during the alignment processes. It means that if we use the same alignment marks on successive alignment steps, then we can neglect mechanical error effects. Thus, for the first EBL step, we only draw alignment marks on the substrate without any patterns. The first layer pattern locates based on these alignment marks. Of course, the relative position between the pattern and the alignment marks don’t perfectly match with the mask design because the stage movement introduces some errors, i.e. the mechanical errors. However, as the following overlay steps contain the same mechanical errors, the effect of the mechanical errors between the first layer pattern and second layer pattern are removed. Using this method, sub-20 nm alignment errors with relatively high reproducibility are achieved. In this work, all the examples have been demonstrated through this method.

### Advanced 3D nanostructures realized by modified EBL overlay process

**Figure 5:**
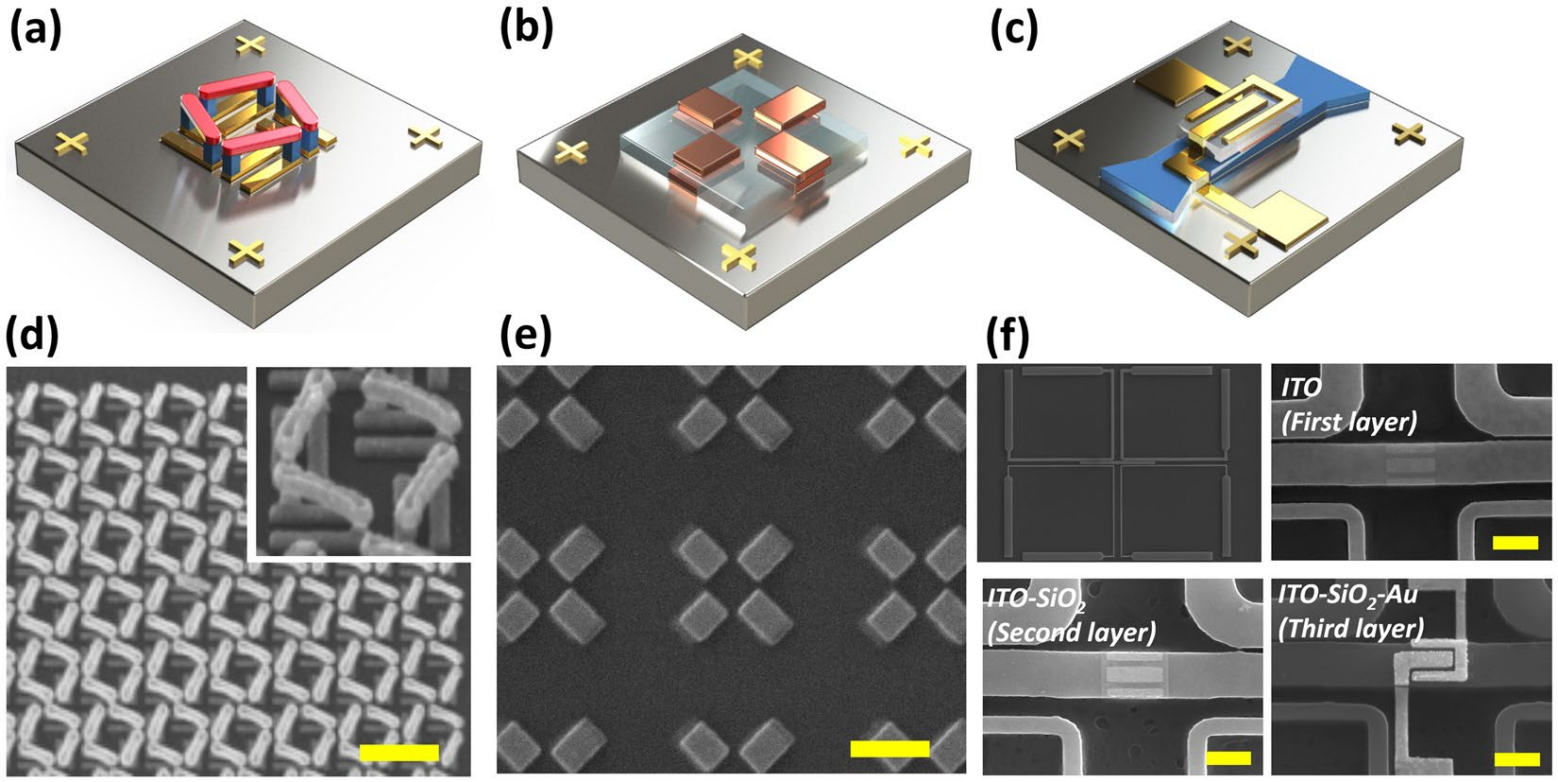
Fabricated 3D nanostructures realized with the proposed EBL overlay process. Suspended/connected, interlayered and hierarchical 3D nanostructures or nanodevices are shown. (**a**) 3D suspended and connected chiral nanostructure which is composed of Au. Upper bars are supported by the middle pillars. (**b**) 3D interlayered toroidal nanostructure for an artificial toroidal dipole response at NIR. Au is used for the nanostructures and SiO_2_ is used for spacer. (**c**) 3D hierarchical structured waveguide device. On top of the waveguide, three metal-oxide-semiconductor (Au-SiO_2_-Indium tin oxide (ITO)) structures are stacked. Applying a voltage at different parts of the gold electrodes, ITO charge concentrations are changed and specific electric field propagating the waveguide are absorbed. (See the section 3 in Supplementary Information) (**d**–**f**) SEM images of the corresponding 3D nanostructures demonstrated with less than 20 nm misalignment. All scale bars represent 500 nm.

In this work, three types of 3D nanostructures, motivated by metamaterials and plasmonic structures working at optical frequencies which have been demonstrated to achieve the designated extraordinary optical properties^23–32^, are fabricated by EBL overlay process for further studies of fabricated metamaterials and plasmonic structures. (Fig. 5) Although those structures have not been characterized and the expected optical properties have not been measured yet, numerical simulation results show that they are expected to have interesting optical properties at optical frequency ranges (visible-NIR region). The detailed simulation results and further information are shown in Supplementary Information. The first one is the chirality metamaterials. Chirality systems are distinguishable system from their mirror systems and these interesting systems or objects can be easily found in nature. The chirality concept has been adopted in metamaterials researches. For instance, conventional negative index metamaterials (NIM) requires simultaneous negative permittivity and negative permeability^33–37^, but a chiral NIM can achieve the negative index without those constraints. The strong chirality, which is induced from electric and magnetic dipole aligned in the same direction, causes dramatic refractive index changes in the different circular polarized light states. In other words, refractive index for one circularly polarized light increases and for the other case decreases. Microscale NIM induced by chirality was demonstrated in 2008, but its working frequency stayed at THz frequencies because of the size of the unit cell^38^. In this work, in order to realize artificial chirality at higher frequencies, we succeed in shrinking the previously demonstrated structure down to a size 100 times smaller which is expected to have artificial chirality around 2.1 μm to 2.5 μm NIR wavelengths. (Figure S1) Through the demonstrated 3D chiral metamaterials, we are certain that the EBL overlay process can be applied to fabricate genuine suspended and connected 3D nanostructures. Manufacturing interlayered 3D nanostructures is also possible. Second, we also propose a toroid-like nanostructure which is composed of two gold-patterned layers separated by a dielectric spacer. From the simulation result, a strong confinement of electric field and toroidal dipolar response are shown in NIR region. (Figure S2) Such an electromagnetic concentration capability can be applied to studying nonlinear optical phenomena, optical nanocavities and nanoscale optical sensing^39^. The last example is a hierarchical 3D nanostructure whose design is proposed for a mode selective waveguide device^40^. Upon the microscale silicon waveguides, three nanoscale metal-oxide-semiconductor capacitors lie. This waveguide structure is designed to control the mode propagation by selectively absorbing certain modes at the interface between the semiconductor and oxide layers. We use indium-tin-oxide (ITO) and ITO layer characteristics that can be tuned by an external voltage, i.e. insulator-like to metallic. Alternatively, the EBL overlay process can be applied to complex hybrid 2D nanostructures, which cannot be made by a single EBL process. (Figure S3) In conclusion, many different types of 3D nanostructures are still necessary for investigating intriguing nanophotonics research, especially at optical frequencies, and we believe our approach can play a critical role in the research field to realize complex 3D nanostructures^41^.

## Discussion

We investigate building complex 3D nanostructures based on accurate EBL overlay process. The basic idea is stacking individual 2D nanopatterns, and thus alignment errors between each 2D layer determine the quality of the whole structure. To reduce the alignment errors, we define two main factors, i.e. alignment marks deformation and mechanical errors. Alignment mark configurations are revised to minimize deformation caused by the proximity effect. In the case of mechanical errors, we can measure and compensate for the errors by using a special ruler structure, called calibrator, but we invent the strategic way to compensate the errors directly. By defining the alignment marks first, alignment errors between each 2D layer can be reduced even though their absolute position is not correct. Three types of 3D nanostructures whose designs come from metamaterials have been demonstrated to verify the feasibility of the suggested method, and finally we realize the 3D elaborate nanostructures. Further, since the developed EBL overlay process is fully compatible with existing conventional micro/nanoscale fabrication methods, this technique can be useful for a variety of research fields including, but not limited to, metamaterials, plasmonics, nanophotonics and nanoelectronics.

## Methods

### Standard EBL process with double resists

A multi-step EBL (Elionix ELS-7800) process is used for fabricating 3D nanostructures. We use two kinds of e-beam resist, polymethyl methacrylate (Microchem 495 PMMA A2) and copolymer (Microchem MMA (8.5) MAA EL8), in order to generate an undercut profile which can reduce side wall deposition during the later deposition process. The copolymer resist is spin coated at 5,000 rpm for 60 seconds (250 nm thickness) and then baked at 150 °C for 5 minutes. On top of the copolymer layer, the PMMA resist is also spin coated at 2000 rpm for 60 seconds (60 nm thickness) and baked at 180 °C for 5 minutes. Electron beam exposure dose is between 1,200~1,600 μC/cm^2^ depending on the pattern shape. After the exposure step, a develop process is done in the MIBK/IPA = 1:3 solution for 10 minutes at 4 °C, followed by electron beam deposition process of desired materials. Finally, a deposited sample is immersed in hot acetone to remove the unwanted area. The aforementioned conditions are common preparations for single EBL processes defining 2D patterns (Figure S4).

### Fabrication of a 3D suspended and connected nanostructure

The 3D suspended and connected structure is fabricated through triple EBL overlay steps due to its configuration, i.e. first pads, second pillars and third pads. All the structures are composed of Au. The first and third Au pads have 40 nm width and 200 nm length. The second gold pillars have 30 nm width and 40 nm height.

### Fabrication of a 3D interlayered nanostructure

The 3D interlayered structure is composed of three layers. We start with defining 30 nm thick Au bar structures via EBL process and deposit 100 nm thick SiO_2_ spacer layer. Then, we do overlay process for the second layer of 30 nm thick Au bar structures.

### Fabrication of a 3D hierarchical nanostructure

On a silicon-on-insulator (SOI) wafer (260 nm silicon and 2 μm buried oxide), firstly we define waveguide patterns via EBL process and deposit Cr 50 nm acting as an etching mask. After the lift-off process, ICP dry etching (DMS Silicon/metal hybrid etcher) is used to etch 260 nm thick silicon. Cr is removed by a chromium etchant. On top of the waveguide, 10 nm thick ITO patterns are fabricated by EBL overlay and annealed at 250 °C for 2 hours. Then, 20 nm thick SiO_2_ patterns are fabricated by EBL overlay to insulate the ITO layers. Finally, Au electrode patterns are fabricated by EBL overlay process. Each overlay process exhibit sub-20 nm alignment errors.

## Electronic supplementary material

Supplementary Information
