# Supplementary material for: Fabrication of three-dimensional suspended, interlayered and hierarchical nanostructures by accuracy-improved electron beam lithography overlay
Source: Sci Rep. 2017 Jul 27;7:6668. doi: 10.1038/s41598-017-06833-5 (PMC5532261; doi:10.1038/s41598-017-06833-5)
Supplement: Supplementary file 1 — Supplementary Information [file 41598_2017_6833_MOESM1_ESM.pdf]

Supplementary Information

Fabrication of three-dimensional suspended, interlayered and hierarchical nanostructures by accuracy-improved electron beam lithography overlay

Gwanho Yoon[1,†], Inki Kim[1,†], Sunae So[1], Jungho Mun[2], Minkyung Kim[1] and Junsuk Rho[1,2,3,*]

[1]Department of Mechanical Engineering, Pohang University of Science and Technology (POSTECH), Pohang 37673, Republic of Korea

[2]Department of Chemical Engineering, Pohang University of Science and Technology (POSTECH), Pohang 37673, Republic of Korea

[3]National Institute of Nanomaterials Technology (NINT), Pohang 37673, Republic of Korea

[*]correspondence to jsrho@postech.ac.kr

[†]These authors contributed equally to this work.

1. Design and simulation of chirality inducing negative index at near-infrared frequencies in a 3D suspended and connected nanostructures

The demonstrated three-dimensional (3D) chiral nanostructures can exhibit artificial chirality at near-infrared (NIR) regime. Detail geometries information is described in figure S1(a). The artificial chirality characteristic is examined by calculated optical activity (θ) and ellipticity angle (η), meaning rotation of the polarization plane and transmission difference depending on the left/right circularly polarized light, respectively[1].

$$\theta = \frac{\arg(T_+) - \arg(T_-)}{2}, \quad \eta = \arctan[\frac{(|T_+| - |T_-|)}{(|T_+| + |T_-|)}]$$

The strong chirality, which is induced by simultaneous excited electric and magnetic dipoles, can cause negative index in the artificial medium[2,3]. In our structures, distinct chirality occurs from 2.1 μm to 2.5 μm (NIR regime) and this chirality may lead negative refractive index at the corresponding frequencies.

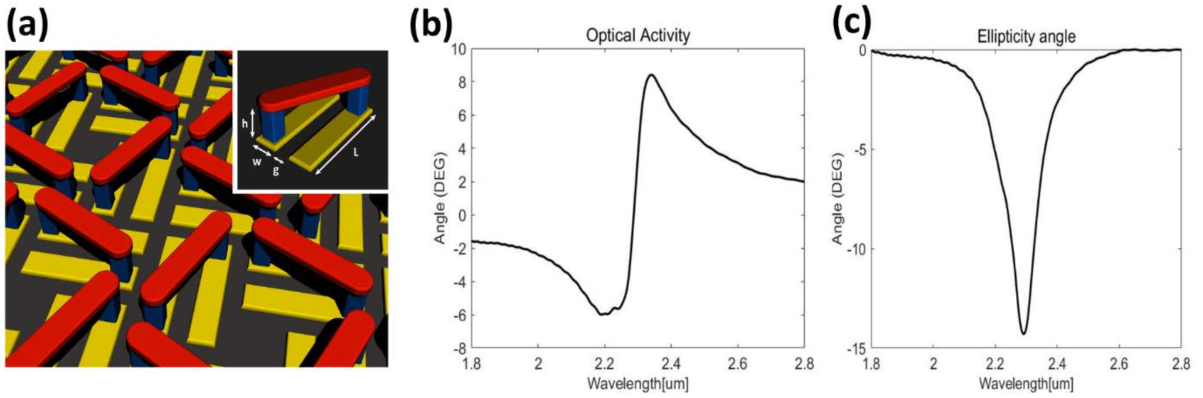

Figure S1. Artificial chirality simulation at NIR frequencies. (a) Schematic of suspended and connected 3D chiral nanostructure which is composed of Au working at optical frequency. Upper bars are supported by the middle pillars. (inset) Specific dimension of the unit cell. L = 200 nm, w = 50 nm, g = 20 nm and h = 40 nm. Glass substrate is used. (b) Calculated optical activity and (c) the ellipticity angle.

2. Design and simulation of artificial optical toroidal dipolar response in a 3D interlayered nanostructure

The interlayered structures are composed of asymmetric four gold (Au) nanobars in bottom and top layers separated by spacer and detail geometries information is described in figure 2S(a). A toroidal dipolar response is a distinctive electromagnetic excitation, which is caused by rotating magnetic field loop. In our proposed structures, electric vortex field is generated at the spacing layer and the electric vortex field induce a magnetic field in the xy-plane which is parallel to the substrate. The induced magnetic fields have different directions at the left and right side structures and those fields make rotating magnetic field loop in the xy-plane. As a result, artificial toroidal excitations are realized by the interlayered structures at a NIR frequency (1.5 μm).

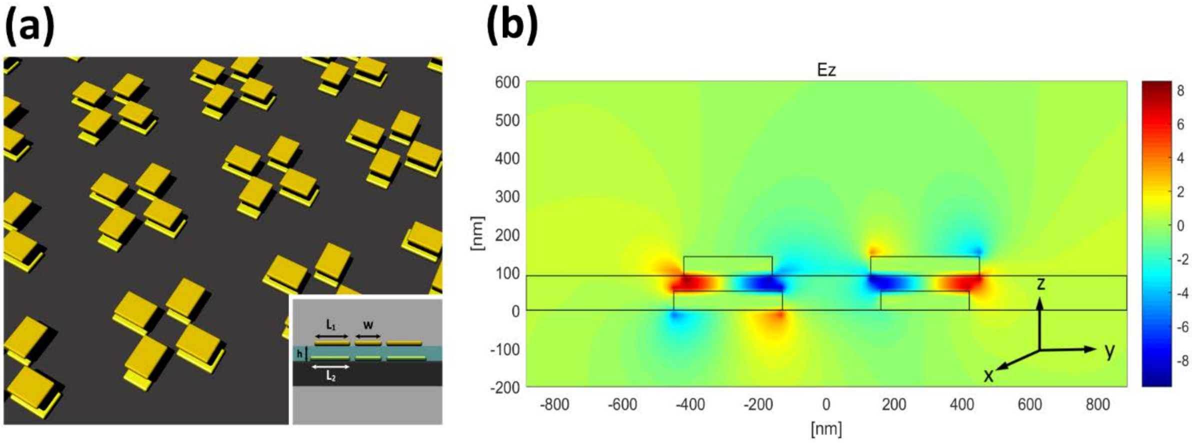

Figure S2. Artificial toroidal dipolar response simulation at NIR frequency. (a) Schematic for the interlayered 3D nanostructures. 30 nm thick Au is used for the nanostructures and SiO_2 is used for spacer. (inset) A cross section view of the unit cell. L_1 = 300 nm, L_2 = 350 nm, w = 200 nm and h = 100 nm. (b) Cross-sectional view of electric field distributions at 1.5 μm. The electromagnetic field is incident on the substrate side.

3. Design and simulation of modal dichroism in a 3D hierarchical nanostructure

The hierarchical structures are composed of microscale silicon waveguide and three nanoscale metal-oxide-semiconductor (MOS) capacitors. On top of the silicon waveguide, using electron-beam lithography (EBL) overlay process, we firstly made indium-tin-oxide (ITO) structures, and then silicon dioxide structures are fabricated as an insulating layers between ITO and top Au electrodes. Finally, Au electrode patterns are fabricated. By applying voltage on the top electrodes, we can tune the charge concentrations of ITO layers, or electric permittivity. Without voltage, ITO acts like insulator, but once voltage is applied to the layer, ITO shows metallic behaviors. Thus, if we apply a proper voltage, then we can maximize imaginary part of permittivity. In other words, ITO patterns can effectively absorb electromagnetic fields. Using this principle, by applying voltage at the certain electrodes, we can selectively absorb a particular mode passing through the waveguides. We call it modal dichroism. The detailed theoretical study for this ITO based modal dichroism modulators was published in the corresponding author' previous publication[4].

4. Design and fabrication of 2D hybrid nanostructures

The proposed EBL overlay process can also be applied to fabricate two-dimensional (2D) and yet more complex hybrid nanostructures, which cannot be made in a single EBL process. Based on such 2D hybrid nanostructures, people have paved the way for deeper understanding of light and matter interactions, including, but not limited to, hydrogen sensing[5], plasmonic color printing[6], magneto-plasmonic interactions[7], chiral plasmonics[8,9] and acousto-plasmonic interactions[10,11]. We demonstrate two kinds of hybrid nanoantennas in order to show the applicability of EBL overlay process in the unconventional hybrid nanoantenna structures. The first example is Au and aluminum (Al) combined nanorods array where 10 nm alignment error exhibits in plane, and the other is a hybrid nanoantennas array consisting of Au/magnesium fluoride (MgF_2) and Au nanorods with two different heights. (Figure S3) The relative position (gap and offset) of each nanorod is controlled accurately and precisely.

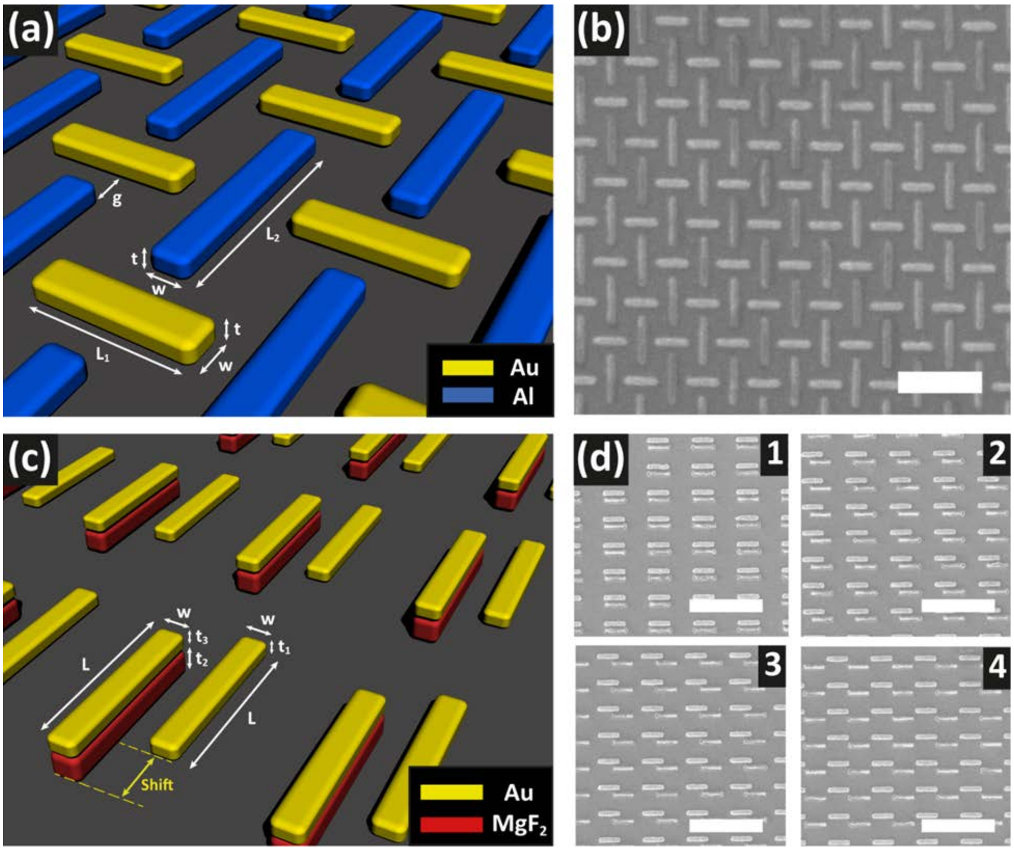

Figure S3. Demonstrated complex hybrid nanostructures consisting of different materials and different heights. (a) Combination of Au and Al nanorods which are perpendicular to each other. L_1 = 200 nm, L_2 = 300 nm, w = 50 nm, t = 30 nm and g = 50 nm. (b) A SEM image of Au and Al nanorods. Different materials give different contrast. Scale bar is 500 nm. (c) Parallel nanorods composed of Au only (lower height) and Au/MgF_2 (higher height). L = 300 nm, w = 50 nm, d=20 nm t_1 = t_3 = 30 nm and t_2 = 65 nm. (d) SEM images of four different alignment configurations. Shift values are 0 nm, 100 nm, 200 nm and 300 nm in the order which is horizontal alignment tolerance, and the gap between two bars is 20nm which is vertical alignment tolerance. All scale bars represent 500 nm.

5. Detailed schematic of the standard EBL process with double resists

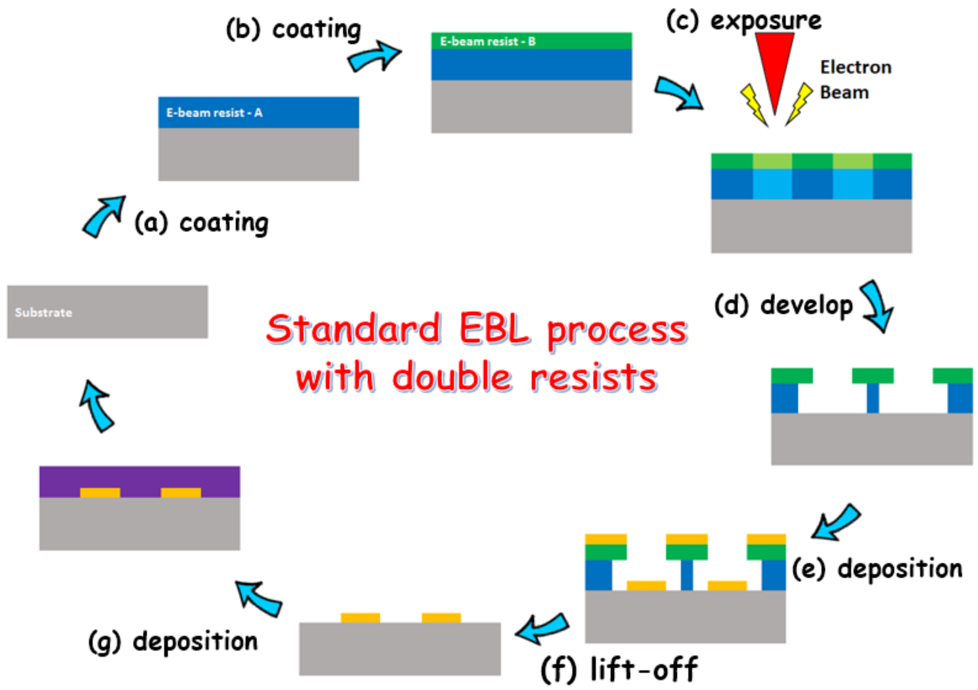

Figure S4. Detailed schematic of the standard EBL process with double resists. (a), (b) Spin coating of the resists with baking. The resist B is the actual patterning resist while the resist A is acting as a spacer for better lift-off process. (c) Electron beam exposing step. Both resists are exposed by the electron beam simultaneously, and the optimal dose value strongly depends on the pattern shape. (d) Since we have used positive resists, exposed area is removed by appropriate develop solution. At this moment, lower resist has higher development rate than upper one so that we can create undercut profile of the resists. Configuration of upper resist determines the final pattern shape. This kind of profile is useful to avoid side-wall effect in a deposition process. (e) Material deposition step. Both sputtering and evaporation can be used for deposition, but evaporation is more feasible than sputtering because it has less side-wall deposition than sputtering. (f) Standard lift-off process. If we put the deposited sample into acetone, unwanted area of the sample is removed owing to dissolution of the remained resist. The side-wall effect plays a critical role in this process because it can interrupt the lift-off process.

References

[1] Li, Z., Mehmet, M. and Ekmel, O. “Chiral metamaterials: from optical activity and negative refractive index to asymmetric transmission.” *J. Opt.* **15**, 023001 (2013).

[2] Pendry, J.B., et al. "A chiral route to negative refraction." *Science* **306**, 1353-1355 (2004).

[3] Zhang, S., et al. "Negative refractive index in chiral metamaterials." *Phys. Rev. Lett.* **102**, 023901 (2009).

[4] Das, S., et al. "Nanophotonic modal dichroism: mode-multiplexed modulators." *Opt. Lett.* **41**, 4394-4397 (2016).

[5] Liu, N., et al. “Nanoantenna-enhanced gas sensing in a single tailored nanofocus.” *Nat. Mater.* **10**, 631-636 (2011).

[6] Duan, X., Kamin, S. and Liu, N. “Dynamic plasmonic colour display.” *Nat. Commun.* **8**, 14606 (2017)

[7] Armelles, G., et al. "Interaction effects between magnetic and chiral building blocks: A new route for tunable magneto-chiral plasmonic structures." *ACS Photonics* **2**, 1272-1277 (2015).

[8] Yin, X., et al. "Interpreting chiral nanophotonic spectra: the plasmonic Born–Kuhn model." *Nano Lett.* **13**, 6238-6243 (2013).

[9] Yin, X., et al. "Active chiral plasmonics." *Nano Lett.* **15**, 4255-4260 (2015).

[10] O’Brien, K., et al. "Ultrafast acousto-plasmonic control and sensing in complex nanostructures." *Nat. Commun.* **5**, 4042 (2014).

[11] Della Picca, F., et al. "Tailored hypersound generation in single plasmonic nanoa ntennas." *Nano Lett.* **16**, 1428-1434 (2016).
